# A novel multi-epitope recombined protein for diagnosis of human brucellosis

**DOI:** 10.1186/s12879-016-1552-9

**Published:** 2016-05-21

**Authors:** Dehui Yin, Li Li, Xiuling Song, Han Li, Juan Wang, Wen Ju, Xiaofeng Qu, Dandan Song, Yushen Liu, Xiangjun Meng, Hongqian Cao, Weiyi Song, Rizeng Meng, Jinhua Liu, Juan Li, Kun Xu

**Affiliations:** Department of Health Laboratory, School of Public Health, Jilin University, Changchun, China; Department of Infection Control, First Hospital of Jilin University, Changchun, China; Jilin Entry-Exit Inspection and Quarantine Bureau, Changchun, China

**Keywords:** Brucellosis, Diagnosis, Recombinant protein

## Abstract

**Background:**

In epidemic regions of the world, brucellosis is a reemerging zoonosis with minimal mortality but is a serious public hygiene problem. Currently, there are various methods for brucellosis diagnosis, however few of them are available to be used to diagnose, especially for serious cross-reaction with other bacteria.

**Method:**

To overcome this disadvantage, we explored a novel multi-epitope recombinant protein as human brucellosis diagnostic antigen. We established an indirect enzyme-linked immunosorbent assay (ELISA) based on this recombinant protein. 248 sera obtained from three different groups including patients with brucellosis (146 samples), non-brucellosis patients (82 samples), and healthy individuals (20 samples) were tested by indirect ELISA. To evaluate the assay, a receiver-operating characteristic (ROC) analysis and immunoblotting were carried out using these characterized serum samples.

**Results:**

For this test, the area under the ROC curve was 0.9409 (95 % confidence interval, 0.9108 to 0.9709), and a sensitivity of 88.89 % and a specificity of 85.54 % was given with a cutoff value of 0.3865 from this ROC analysis. The Western blot results indicate that it is feasible to differentiate human brucellosis and non-brucellosis with the newly established method based on this recombinant protein.

**Conclusion:**

Our results obtained high diagnostic accuracy of the ELISA assay which encourage the use of this novel recombinant protein as diagnostic antigen to implement serological diagnosis of brucellosis.

**Electronic supplementary material:**

The online version of this article (doi:10.1186/s12879-016-1552-9) contains supplementary material, which is available to authorized users.

## Background

*Brucella* spp. are Gram-negative, facultative, intracellular bacterial pathogens that cause brucellosis, an infectious disease affecting animals and humans [1]. Based on the difference in pathogenicity and host preference, three main human brucellosis pathogens, *B. melitensis*, *B. abortus*, and *B. suis* (whose preferred natural host animals are sheep and goats, cattle, and swine, respectively), can infect humans, involving any organ or system of the body, and lead to serious complications with important public health issues [2]. The infection is primarily transmitted by consumption of unpasteurized dairy products, direct contact with infected animals, handling of cultures or clinical specimens. The disease remains endemic in many regions of the world, including Latin America, the Middle East, Africa, Asia, and the Mediterranean basin, affecting approximately 500,000 people annually around the world [3]. As the largest developing country in the world, the incidence of human brucellosis has rapidly increased in China since 1995 [4]. According to data from the Chinese Center for Disease Control and Prevention (CDC), more than 57,000 human cases were identified in 2014 (http://www.nhfpc.gov.cn/jkj /s3578/ 201502/847c041a3bac4c3e844f17309be0cabd.shtml). According to the previous study, brucellosis is mainly distributed in some of the northern provinces of China, accounted for >90 % of the reported cases. Jilin province has the fourth highest incidence, with the annual incidence ranging from 50 to 100 per 1000,000 [5].

Because of a deficiency of clinical pathognomonic symptoms, a prompt and accurate diagnosis is important. Current methods used for identification of brucellosis include traditional culture-, immunological-, and molecular-based methods, which usually follow a bacterial enrichment step. Blood culture, which was considered the golden standard method, provides the definitive diagnosis of brucellosis but may not provide a positive result for all patients [6]; it presents several other drawbacks, such as being time-consuming and dangerous for personnel, and few laboratories have suitable culture conditions [7]. Although polymerase chain reaction (PCR) and real-time PCR assays are very promising, infrastructure, equipment, and expertise are lacking in developing countries [8].Therefore, serological tests, such as the enzyme-linked immunosorbent assay (ELISA) and standard tube agglutination test (SAT), have become the most useful tools for diagnosis of brucellosis. In particular, ELISA can provide higher sensitivity and specificity compared with SAT [9]. The crucial part of ELISAs is a sensitive and specific diagnostic antigen. Several immunogenic *Brucella* spp. surface-exposed outer membrane proteins (OMPs), such as OMP16 [10], OMP25 [11], OMP2b [12], and OMP31 [13], and periplasmic protein 26 (BP26) [14] have been previously identified, which indicates these immunoreactive proteins are potential candidates of diagnostic antigens for ELISAs.

Currently, in endemic areas, many countries have developed control measures for eradicating the disease in livestock animals. Vaccination is probably the most common measure for controlling brucellosis; live attenuated vaccines were used. Unfortunately, because the vaccines could cause animal abortion, infertility, weak offspring, and other shortcomings their application was blocked [15]. In order to overcome these disadvantages, researchers are trying to develop new vaccines, and recombination vaccines were produced [16]. Prediction of antigenic epitopes on protein surfaces is important for vaccine design. Most existing epitope prediction methods focus on protein sequences to predict continuous epitopes linear in sequence. These prediction methods are based upon the amino acid properties, including immunoinformatic analysis and prediction of B-cell epitopes, such as hydrophilicity [17], surface accessibility [18], secondary structure [19], flexibility [20], ABCPred [21], COBEPro [22], and BepiPred [23]. With the development of these bioinformatics methods, they provide more cost-effective approaches for seeking *Brucella* vaccines and brucellosis diagnostic antigens.

For diagnose specificity, monoclonal antibodies are the best option, but a monoclonal antibody can recognize only one unique epitope. The preparation of monoclonal antibodies is time-consuming and laborious, and whether monoclonal antibodies can be prepared in large batches depends on the luck component. In the present work, we used bioinformatic methods for B-cell epitope prediction to predict B-cell epitopes of OMP16, OMP2b, OMP31, and BP26; based on these predicted B-cell epitopes, we designed a novel recombinant protein for serological diagnostic of brucellosis. We expressed and purified this recombinant protein and finally evaluated it for its diagnostic utility with ELISA and Western blotting. Our design can avoid the shortcomings of monoclonal antibodies.

## Methods

### Computer modeling prediction of epitopes

Amino acid sequences of OMP16, OMP2b, OMP31, and BP26 were obtained from the National Center for Biotechnology Information (NCBI) Database at http://www.ncbi.nlm.nih.gov. To ensure the accuracy of prediction, three different epitope prediction software programs based on the protein sequences were used to predict the most immunogenic linear B-cell epitopes: Bepipred (http://www.cbs.dtu.dk/services/Bepipred/), ABCpred (http://www.imtech.res.in/raghava/abcpred/), and COBEpro (http://scratch.proteomics.ics.uci.edu/). To find the presence or absence of predicted epitopes across species, the BLASTP was performed.

### Construction, expression, and purification of recombinant proteins

First, the predicted B-cell epitopes were selected to construct the recombinant outer membrane protein(rOmp). Then adjacent epitopes were joined together by the linker “GGGS.” The recombinant protein gene was synthesized by Sangon Biotech (Shanghai, China), which had a six-His-tag-encoding sequence at the 3´ end, by using codon optimized to permit expression in *Escherichia coli*. The synthetic gene was inserted in the bacterial expression vector pET-28b (+) to generate the plasmid pET28b (+); then the recombinant plasmid was transformed into *E. coli* BL21 (DE3) cells to induce recombinant protein expression with 0.2 mM isopropyl-β-D-thiogalactoside (IPTG).

The transformed *E. coli* BL21 cells were grown in a shaker at 220 rpm and 37 °C, inoculated into 4 L of Luria-Bertani (LB, containing 30 μg/ml kanamycin) at a dilution of 1:100. When the optical density at 600 nm (OD600) of the culture reached approximately 0.6, the culture was induced by 0.2 mM IPTG. Induction was grown overnight, and cells were collected by centrifugation. Aliquots of the induced and noninduced cell cultures were analyzed by SDS-PAGE prior to purification.

The collected cells were dissolved with a lysis buffer (7 M guanidine hydrochloride, 50 mM Tris, 300 mM NaCl, 0.1 % Triton X-100, DNase I, RNase, lysozyme, pH = 8.0) and then homogenized by sonication on ice (power 400 W, 20 min, ultrasonic 3 S, pause 5 S for a loop). The lysate was clarified by centrifugation at 10,000 × g for 20 min at 4 °C; then the supernatant was collected for purification. The supernatant was gently shaken with 5 ml of Ni-nitrilotriacetic acid (NTA). The column was pre-equilibrated with a binding buffer (7 M Gua-Hcl, 50 mM Tris, 300 mM NaCl, pH = 8.0). After the flow-through was collected, the column was washed extensively with wash buffer (8 M urea, 50 mM Tris, 300 mM NaCl, 10/20/50 mM imidazole, pH = 8.0) and elution buffer eluent (8 M urea, 50 mM Tris, 300 mM NaCl, 500 mM imidazole, pH = 8.0); the eluent was collected.

The purified rOmp protein was run on 12 % SDS-PAGE gel and transferred to a polyvinylidene fluoride (PVDF) membrane in a trans-blot system. After the transfer, the membrane was blocked with 5 % skim milk at 4 °C overnight, and then washed three times with phosphate-buffered saline containing 0.05 % Tween 20 (PBST) and incubated with a rabbit anti-His-tag polyclonal antibody (at 1:1000 dilution) for 1 h at 37 °C. The membrane was washed three times again, and incubated with horseradish peroxidase (HRP)-conjugated goat anti-rabbit immunoglobulin (Ig)G antibodies at 1:10,000 dilutions for 1 h at 37 °C. After washing again, the protein bands were incubated in diaminobenzidine (DAB) substrate solution for 15 min at 37 °C.

Concentrations of purified proteins were measured by non-interference protein assay kit SK3071 (Sangon Biotech,Shanghai, China), according to the instructions supplied by the kit manufacturer.

### Samples

248 serum samples from three different clinical groups were provided by Plague and Brucellosis Prevention and Control Base, Chinese CDC, Baicheng, Jilin, China:

1.  146 serum brucellosis samples of culture-positive, which were serologically positive by plate agglutination test (PAT) and standard tube agglutination test (SAT) as standard methods for diagnosis of brucellosis.
2.  82 serum samples of non-brucellosis infected other bacteria, including *E. coli* (5 serum samples), *Staphylococcus* (10 serum samples), *Proteus mirabilis* (2 serum samples), *Enterobacter cloacae* (2 serum samples), *Streptococcus salivarius* (2 serum samples), *Streptococcus viridans* (2 serum samples), and *Klebsiella pneumoniae* (6 serum samples), *Salmonella enteritidis* (1 serum samples), and fever of unknown origin (62 serum samples).
3.  20 serum samples of healthy individuals were collected. The samples of non-brucellosis patients and healthy donors were verified as non-brucellosis by PAT and SAT.

Venous blood (5 ml) was collected from the patients and healthy individuals; supernatants were harvested after centrifugation at 1,200 g for 10 min and stored at −70 °C until assayed. A titer of 1:100 and higher was considered as a suspected positive result for brucellosis by PAT or SAT, and blood culture was subsequently used for verification.

### IELISA and Western blot analysis

Ninety-six-well microtiter plates (Corning, USA) were coated with 1 μg per well of the recombinant protein in coating buffer (0.01 M PBS, pH 7.4) and incubated overnight at 4 °C. After three washes with PBST, the plates were blocked with 1 % bovine serum albumin (BSA, Sigma, USA) for 2 h at 37 °C and then incubated with serum 1:400 in PBST containing 1 % BSA for 1 h at 37 °C. After a washing step, a 1:10,000 diluted HRP-goat anti-human IgG secondary antibody (Invitrogen, USA) was added and incubated at 37 °C for 1 h. The plates were washed, and then 100 μl of the TMB substrate solution was added to each well. Reactions were left to react for 15 minutes at room temperature in a dark place. Finally, 50 μl of the stop solution (2 M H2SO4) was added to each well. ODs were measured at 450 nm using an ELISA plate reader (BioTek, USA). Positive, negative, and blank (PBS) samples were tested in triplicate, and the experiment was repeated three times. All the samples were processed simultaneously.

Meanwhile, we used the antigen of SAT (the whole bacteria antigen were supplied by Plague and Brucellosis Prevention and Control Base) to evaluate the effectiveness of our fusion protein as a diagnostic antigen for brucellosis by IELISA.

Western blotting was performed partially as described above. After recombinant proteins were transferred onto the PVDF membrane, the membranes were blocked in TBST containing 5 % skim milk overnight at 4 °C. Then the membranes were incubated with human serum at 1:100 dilution for 2 h at room temperature. After a washing step with TBST, the membranes were incubated with a 1:10,000 HRP-conjugated goat anti-human IgG secondary antibody for 1 h at room temperature (RT). After three washes in TBST, protein detection was visualized with the BeyoECL Plus Kit (Beyotime, China), following the manufacturer's instructions, and exposed to X-ray film. Each band was analyzed using a Gel Image System (Tanon, China).

### Statistical analysis

Dotplot and receiver-operating characteristic (ROC) were performed using the GraphPad Prism version 6.05 for Windows.

## Results

### Computer modeling prediction of epitopes

The Accession Numbers of OMP16, OMP2b, OMP31, and BP26 were obtained from NCBIDatabase(Additional file 1: Table S3). To improve the accuracy of B-cell epitope prediction, three different epitope-prediction software programs (ABCPred, BepiPred, and COBEPro) were used to predict the most immunogenic linear B-cell epitopes of the fusion protein. We choose the 15 overlapping epitopes that were predicted by the three methods as B-cell epitope candidates (see Table 1), and the results of BLASTP indicated these epitopes are highly conserved in *Brucella*.

**Table 1:** Overlapping epitopes predicted by three methods

Protein	Start–end position	Sequence
BP26	87–124	KKAGIEDRDLQTGGINIQPIYVYPDDKNNLKEPTITGY
	151–170	GVNQGGDLNL VNDNPSAVIN
	223–245	AAAPDNSV PIAAGENSYNVSVNV
OMP31	24–40	VSEPSAP TAAPVDTFSW
	48-83	NAGYAGGKFKHPFSSFDKEDNEQVSGSLDVTAGGFV
	109–129	SS VTGSISAGAS GLEGKAETK
	168–225	GDD ASALHTWSD KTKAGWTLGAGA EYAINNNWTLKSEYLYTDLGKRNLVDVDNSFLES
OMP16	90–106	Q YSITIEGHAD ERGTRE
	124–146	ASRGVPT NRMRTISYGN ERPVAV
OMP2b	66–84	DVKGG DDVYSGTDRN GWDK
	194–210	ALEQGGD NDGGYTGTTN
	242–282	VIEEWAAKVRGDVNITDQFSVWLQGAYSSAATPDQNYGQWG
	268–286	YSS AATPDQNYGQ WGGDWA
	306–316	FNLQAAHDDW GKTAVTAN
	328–354	TVT PEVSYTKFGG EWKDTVAEDN AWGG

### Recombinant protein identification

**Fig. 1:**
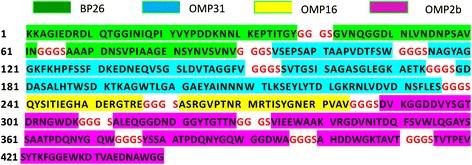
Amino acid sequence of the recombinant protein in FAST format: “GGGS” is the linker

**Fig. 2:**
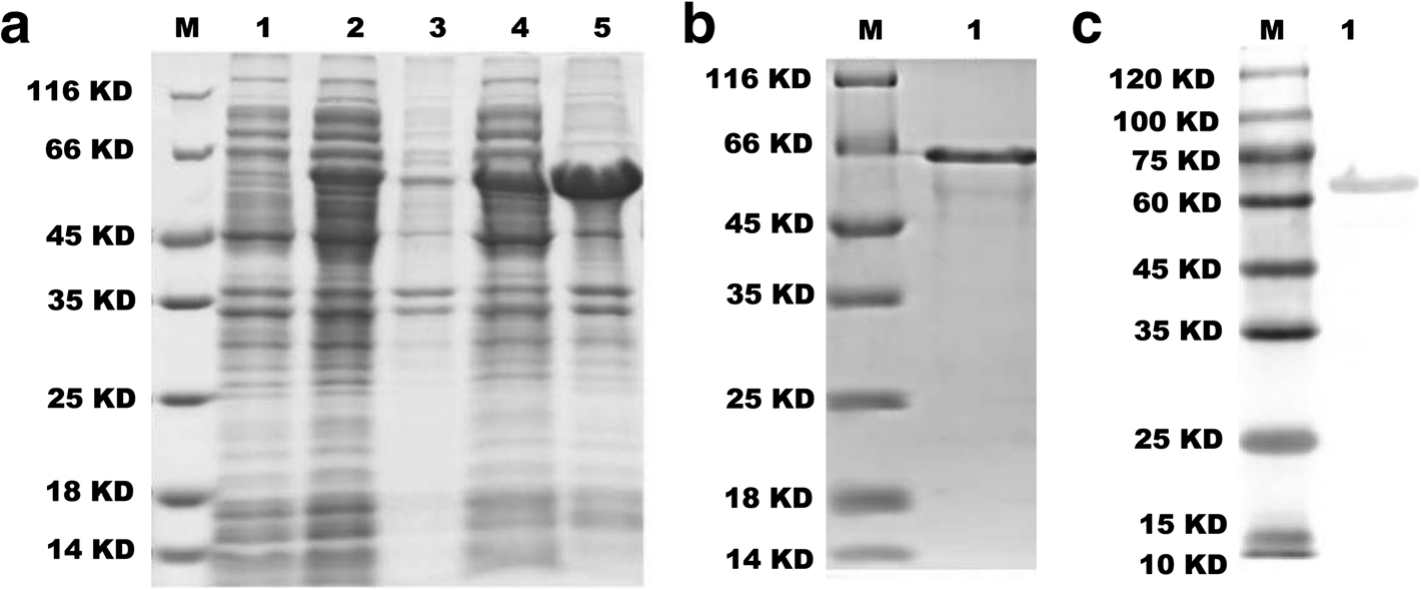
Preparation and identification of recombinant protein. **a** SDS-PAGE of OMP-induced expression with 0.2 mM IPTG for different times (M, marker; Lane 1, uninduced cells; Lane 2, supernatant of IPTG-induced cells for 2 h; Lane 3, deposition of IPTG-induced cells for 2 h; Lane 4, supernatant of IPTG-induced cells for 4 h; Lane 5, deposition of IPTG-induced cells for 4 h). **b** SDS-PAGE of OMP purification (M, marker; Lane 1, purified recombinant protein.). **c** Western blotting analysis of the purified recombinant protein using the anti-His-tag rabbit anti-His-tag polyclonal antibody (M, marker; Lane 1, purified recombinant protein)

To avoid emerging new epitopes, we separated each predicted epitope by a linker (Fig. 1). By optimizing codons for *E. coli* expression, a resultant 1338-bp gene was synthesized by Sangon Biotech. Then we inserted the synthetic gene in the bacterial expression vector pET-28b and transformed it into *E. coli* BL21 (DE3) cells. Then, the induced cells were sonicated and analyzed by SDS-PAGE. A specific band representing the recombinant product was obtained (Fig. 2a). Ni–NTA affinity purification was used, and the purified products were analyzed by SDS-PAGE (Fig. 2b and c). Starting from 1 g of the induced cell pellet, we obtained 1 ml of rOmp protein solution with a concentration of 1.2 mg/ml, as measured with a non-interference protein assay kit (SK3071, Sangon Biotech).

### IELISA

**Fig. 3:**
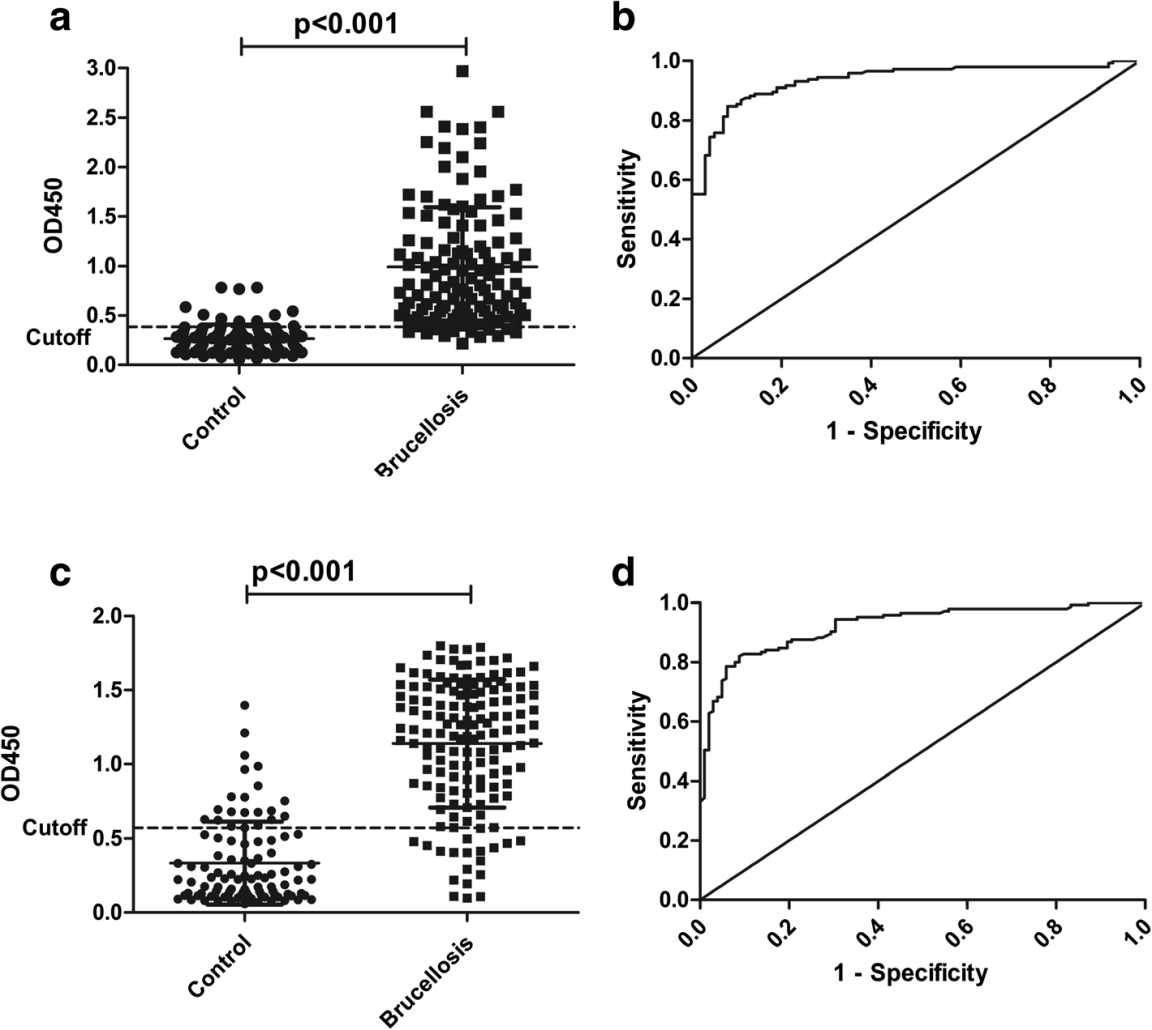
IELISA analysis of serum samples. The analysis was performed considering positive control serum samples with culture-confirmed/serologically positive brucellosis (146 sera) and negative control sera from patients with other diseases and healthy individuals (102 sera). **a** Dotplot of the rOMP IELISA assay. **b** ROC analysis of rOMP IELISA assay results. **c** Dotplot of the SAT antigen IELISA assay results. **d** ROC analysis of SAT antigen IELISA assay results

To evaluate the assay, 248 serum samples including brucellosis (146 samples), non-brucellosis patients (82 samples) and healthy individuals (20 samples) were tested by indirect ELISA and Western blotting. A dotplot diagram outlined the OD values of these samples (Fig. 3a). A ROC analysis was performed to evaluate the optimize sensitivity and specificity (Fig. 3b). Based on the ROC analysis, AUC for this test was 0.9409 (95 % confidence interval (CI), 0.9108 to 0.9709). In addition, a diagnostic sensitivity of 88.89 % (95 % CI, 82.06 to 93.79) and a specificity of 85.54 % (95 % CI, 76.11 to 92.30) was obtained from a optimum cutoff value(0.3865). With this cutoff value, 135 of 146 brucellosis cases were diagnosed correctly as positive, and only nine negative cases were diagnosed incorrectly as positive; all of the healthy individuals can be diagnosed correctly.

Furthermore, we used the antigen of SAT to diagnose brucellosis in IELISA, which was in parallel with the fusion protein, a dotplot diagram (Fig. 3c), and ROC curve (Fig. 3d). Based on same analysis, the AUC for the test was 0.8981 (95 % CI, 0.8455 to 0.9508), a diagnostic sensitivity of 86.99 % (95 % CI, 80.43 to 91.98)and a specificity of 82.05 % (95 % CI, 66.46 to 92.46) was obtained from a optimum cutoff value(0.5730). However, with the cutoff value, 127 of 146 brucellosis cases were diagnosed correctly, and 20 negative cases were diagnosed incorrectly, including seven healthy individuals diagnosed as positive. We also have a cross-table with absolute numbers of positive and negative samples with these cutoff values (Table 2).

**Table 2:** Positive predictive value and negative predictive value of different cutoff values

Cutoff value	Positive		Negative		PPV (%)	NPV (%)
	TP	FN	TN	FP		
≥0.3865^a^	135	11	93	9	93.75	89.42
≥0.5730^b^	127	19	82	20	80.89	81.19

TP, true positives; TN, true negatives; FP, false positives; FN, false negatives; PPV, positive predictive value (TP/TP + FP) × 100; NPV, negative predictive value (TN/TN + FN) × 100

^a^cutoff value is calculated by rOMP ELISA

^b^cutoff value is calculated by antigen of SAT ELISA

The results of ELISA indicate that it is possible with this assay to clearly discriminate between brucellosis and non-brucellosis cases. Compared with the whole bacterial antigen, rOMP has a weaker cross-reaction (Additional file 2: Fig. S1).To further investigate isotype-specific reactivity, IgM, IgG, and Ig(M + G) ELISA tests were performed. We found that the IgG and Ig(M + G) had similar results, but only a few brucellosis samples were weakly IgM isotype positive. Compared with the antigen of SAT, 45 brucellosis samples were IgM isotype positive (Additional file 3: Table S2).

Additionally, the western blotting result indicates that the antibody could specificity binding to the recombinant protein of predicted B-cell epitopes.

## Discussion

B-cell epitopes are the sites of molecules that are recognized by antibodies of the immune system. Bioinformatics is used for many purposes, especially for vaccine design and diagnostic tests. These prediction methods are based upon the properties of 20 amino acids, including immunoinformatic analysis and prediction of B-cell epitopes, such as hydrophilicity [17], surface accessibility [18], β-turn [19], and flexibility [20]. However, using bioinformatic tools to predict immunogenic B-cell epitopes remains a vital and challenging task because their rate of successful prediction is not very high. To avoid the drawbacks, three different epitope prediction software programs (ABCPred, BepiPred, and COBEPro) were used to predict the most immunogenic linear B-cell epitopes in this study.

In the early 1980s and 1990s, the major outer proteins of *Brucella* spp. were identified and characterized as potential immunogenic and protective antigens. In recent studies, several OMPs had been demonstrated to be highly immunoreactive; these include BP26, OMP16, OMP31, OMP2b, and OMP25. In our study, we chose four OMPs (BP26, OMP16, OMP31, and OMP2b), which exist in almost all *Brucella* spp. We did not use OMP25 because it is present mainly in *B. abortus*. We used ABCPred, BepiPred, and COBEPro to predict the most immunogenic linear B-cell epitopes in the four selected OMPs. To avoid inaccuracies, we selected 15 overlapping epitopes that were predicted by all three methods.

It is reported that laboratory testing and diagnosis of brucellosis is critical for patient management [24]. Currently, numerous available diagnostic methods are difficult to perform in developing countries with poor resources, especially in endemic regions. IELISA is the best choice for diagnosing brucellosis, especially when other tests are limited. It can reveal total and individual specific immunoglobulins (IgG, IgM, IgA) rapidly (within 6 h) with high sensitivity and specificity [25].

In our present study, we designed a novel antigen to detect antibodies against *Brucella* spp. using an indirect ELISA assay. The assay was assessed by using a series of serum samples obtained from brucellosis, non-brucellosis and healthy individuals. The sample characteristics are described in Additional file 4: Table S1. All the samples are from Baicheng city, which has a high incidence of brucellosis in China. In this region, almost all farmers breed livestock, which accounts for significant morbidity of brucellosis.

To validate the assay, an immunoblot and ROC analysis were performed. The result of AUC was 0.9409, indicating our assay has high accuracy for human brucellosis diagnose by contrast with global summary statistics of diagnostic accuracy of AUC. Our method could distinguish highly accurate (0.9 < AUC < 1) [26]. Because brucellosis patients often have gastrointestinal symptoms and fever, we chose patients with other bacterial infections as a portion of the controls, such as *E. coli*, *Staphylococcus*, *Salmonella enteritidis*, and fever of unknown origin. These patients are likely to be misdiagnosed. The Western blot results indicate that the newly established method based on this recombinant protein is feasible to differentiate human brucellosis and non-brucellosis infections.

However, ELISA methods that detect IgG are sensitive but have low specificity. Measurement of IgM levels has lower sensitivity than IgG but is more specific [27]. In this case, this recombinant protein has low sensitivity to diagnose brucellosis in acute infection. The other, because of the confirmation in brucellosis reports of China, and indiscriminate therapeutic regimen, the diagnosis of brucellosis often do not distinguish *Brucella* species. We did not obtain any information of diagnostic value for different *Brucella* species infection. However, a previous study has confirmed that *B. melitensis* is the most prominent species causing human brucellosis in China, accounting for more than 80 % of the cases [28].

## Conclusion

Herein, we validated the newly established IELISA assay with a high diagnostic accuracy of human brucellosis. In our study, a predicted recombinant protein from four major OMPs of *Brucella* was expressed for the first time and has been validated to diagnose human brucellosis. However, to fully evaluate the recombinant protein for diagnosis, further research will be necessitated for diagnostics of brucellosis caused by different *Brucella* spp. strains.

In addition,this recombinant protein based assay can be desiged and established for other infectious diseases diagnosis.

### Consent for publication

Not applicable.

### Ethics approval and consent to participate

The serum samples used in this study were obtained for laboratory analysis. Written informed consent for sample analysis was obtained from the patients. This study was approved by the Institutional Research Ethics Committee of Medicine, the School of Public Health, Jilin University, permit number: JLU2014-0303.

### Availability of data and materials

All the data supporting the conclusions of this article is available and included within the article and its additional files.

## Additional files

Additional file 1: Table S3. The Accession Numbers of OMPs of Brucella spp. in NCBI Protein database. (DOC 32 kb) Additional file 2: Fig S1. IELISA assay analysis of serum samples in negative controls, healthy individuals and patients with other bacterial infection. (DOC 1403 kb) Additional file 3: Table S2. Isotype specific reactivity positive results from the sera of brucellosis patients(146 samples) and controls(102 samples) in ELISA. (DOC 30 kb) Additional file 4: Table S1. Specimen characteristics of positive and negative. (DOC 30 kb)

## Abbreviations

ELISA: Enzyme-linked immunosorbent assay
ROC: Receiver-operating characteristic
CDC: Chinese center for disease control and prevention
PCR: Polymerase chain reaction
OMPs: Outer membrane proteins
NCBI: National center for biotechnology information
LB: Luria-Bertani
OD: Optical density
NTA: Ni-nitrilotriacetic acid
PAT: Plate agglutination test
SAT: standard tube agglutination test
PVDF: Polyvinylidene fluoride
RT: Room temperature
PTG: Isopropyl-β-D-thiogalactoside
CI: Confidence interval
rOmp: Recombinant outer membrane protein
HRP: Horseradish peroxidase
BSA: Bovine serum albumin
AUC: The area under the ROC curve
