# Supplementary material for: A novel multi-epitope recombined protein for diagnosis of human brucellosis
Source: BMC Infect Dis. 2016 May 21;16:219. doi: 10.1186/s12879-016-1552-9 (PMC4875615; doi:10.1186/s12879-016-1552-9)
Supplement: Additional file 1: Table S3. — The Accession Numbers of OMPs of Brucella spp. in NCBI Protein database. (DOC 32 kb) [file 12879_2016_1552_MOESM1_ESM.doc]

**Table S3**. The Accession Numbers of OMPs of Brucella spp. in NCBI Protein database

| OMPs | omp31 | bp26 | omp16 | | omp2b |
| --- | --- | --- | --- | --- | --- |
| Accession  Numbers | ACS50328.1 | AAO39775.1 | AAA59360.1 | | AGO95098.1 |
|  | AAL27290.1 | AAO39774.1 | AEF59023.1 | | AKN23426.1 |
|  | AAL27296.1 | AAO39773.1 | | | AHN46277.1 |
|  | AAL27292.1 | AAB38523.1 | |  |  |
