# Supplementary material for: A novel multi-epitope recombined protein for diagnosis of human brucellosis
Source: BMC Infect Dis. 2016 May 21;16:219. doi: 10.1186/s12879-016-1552-9 (PMC4875615; doi:10.1186/s12879-016-1552-9)
Supplement: Additional file 2: Fig S1. — IELISA assay analysis of serum samples in negative controls, healthy individuals and patients with other bacterial infection. (DOC 1403 kb) [file 12879_2016_1552_MOESM2_ESM.doc]

Fig S1. IELISA assay analysis of serum samples in negative controls, healthy individuals and patients with other bacterial infection. A: Dotplot of the rOMP IELISA assay results. B: Dotplot of the SAT antigen IELISA assay results.
