# Supplementary material for: A novel multi-epitope recombined protein for diagnosis of human brucellosis
Source: BMC Infect Dis. 2016 May 21;16:219. doi: 10.1186/s12879-016-1552-9 (PMC4875615; doi:10.1186/s12879-016-1552-9)
Supplement: Additional file 3: Table S2. — Isotype specific reactivity positive results from the sera of brucellosis patients(146 samples) and controls(102 samples) in ELISA. (DOC 30 kb) [file 12879_2016_1552_MOESM3_ESM.doc]

**Table S2.** Isotype specific reactivity positive results from the sera of brucellosis patients(146 samples) and controls(102 samples) in ELISA.

| Group | Brucellosis | | | Controls | | |
| --- | --- | --- | --- | --- | --- | --- |
|  | IgM | IgG | Ig(M+G) | IgM | IgG | Ig(M+G) |
| Romp | 3 | 135 | 135 | 0 | 9 | 9 |
| Antigen of SAT | 46 | 127 | 128 | 1 | 20 | 20 |
