# Supplementary material for: A novel multi-epitope recombined protein for diagnosis of human brucellosis
Source: BMC Infect Dis. 2016 May 21;16:219. doi: 10.1186/s12879-016-1552-9 (PMC4875615; doi:10.1186/s12879-016-1552-9)
Supplement: Additional file 4: Table S1. — Specimen characteristics of positive and negative. (DOC 30 kb) [file 12879_2016_1552_MOESM4_ESM.doc]

**Table S1.** Specimen characteristics of positive and negative.

| Group | | Brucellosis | Non-brucellosis |
| --- | --- | --- | --- |
| Gender | Male | 94(64.39%) | 71(69.61%) |
|  | Female | 52(35.71%) | 31(30.39%) |
| Age |  | 51.58±13.47 | 49.31±10.21 |
| Occupation | Peasant | 132(90.41%) | 92(90.2%) |
|  | Pastoralist | 6(4.11%) | 4(3.92%) |
|  | Other | 8(5.48%) | 6(5.88%) |
